# Correction: Heat-related mortality in U.S. state and private prisons: A case-crossover analysis

**DOI:** 10.1371/journal.pone.0360436

**Published:** 2026-10-09

**Authors:** Julianne Skarha, Keith Spangler, David Dosa, Josiah D. Rich, David A. Savitz, Antonella Zanobetti

In the Abstract section, the third sentence is incorrect. The correct sentence is: Yet, there is scarce literature on the impacts of heat among incarcerated populations.

The images for Figs 1 and 2 are incorrectly switched. The image that appears as Fig 1 should be Fig 2, and the image that appears as Fig 2 should be Fig 1. The figure captions appear in the correct order. The authors have provided a corrected version of the figures here.

**Fig 1:**
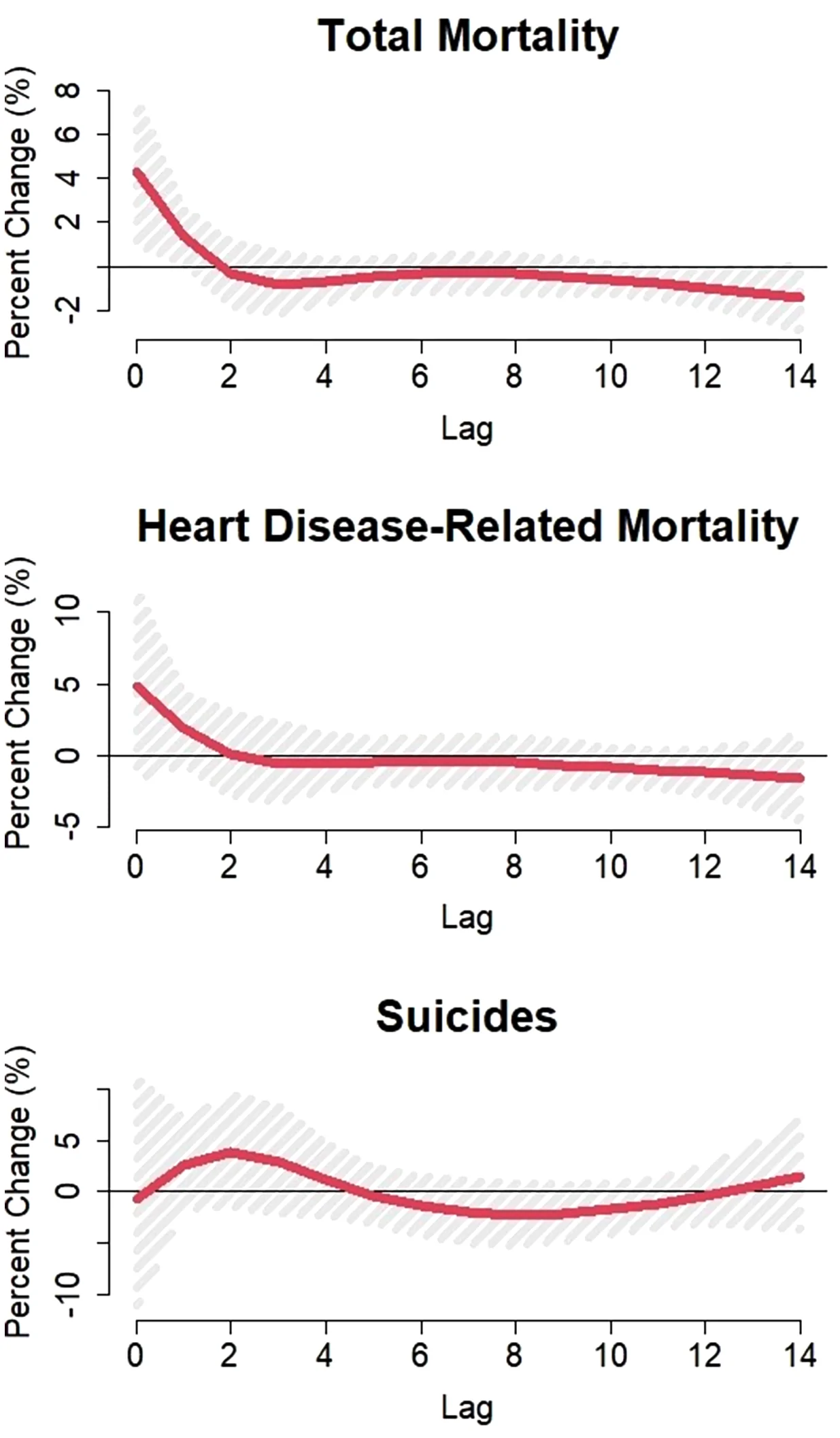
Lag-response curves representing the percent change in total mortality, heart disease-related mortality, and suicides associated with a 10°F degree increase in mean temperature above the prison-specific summer average at each lag in U.S. prisons between 2001–2019.

**Fig 2:**
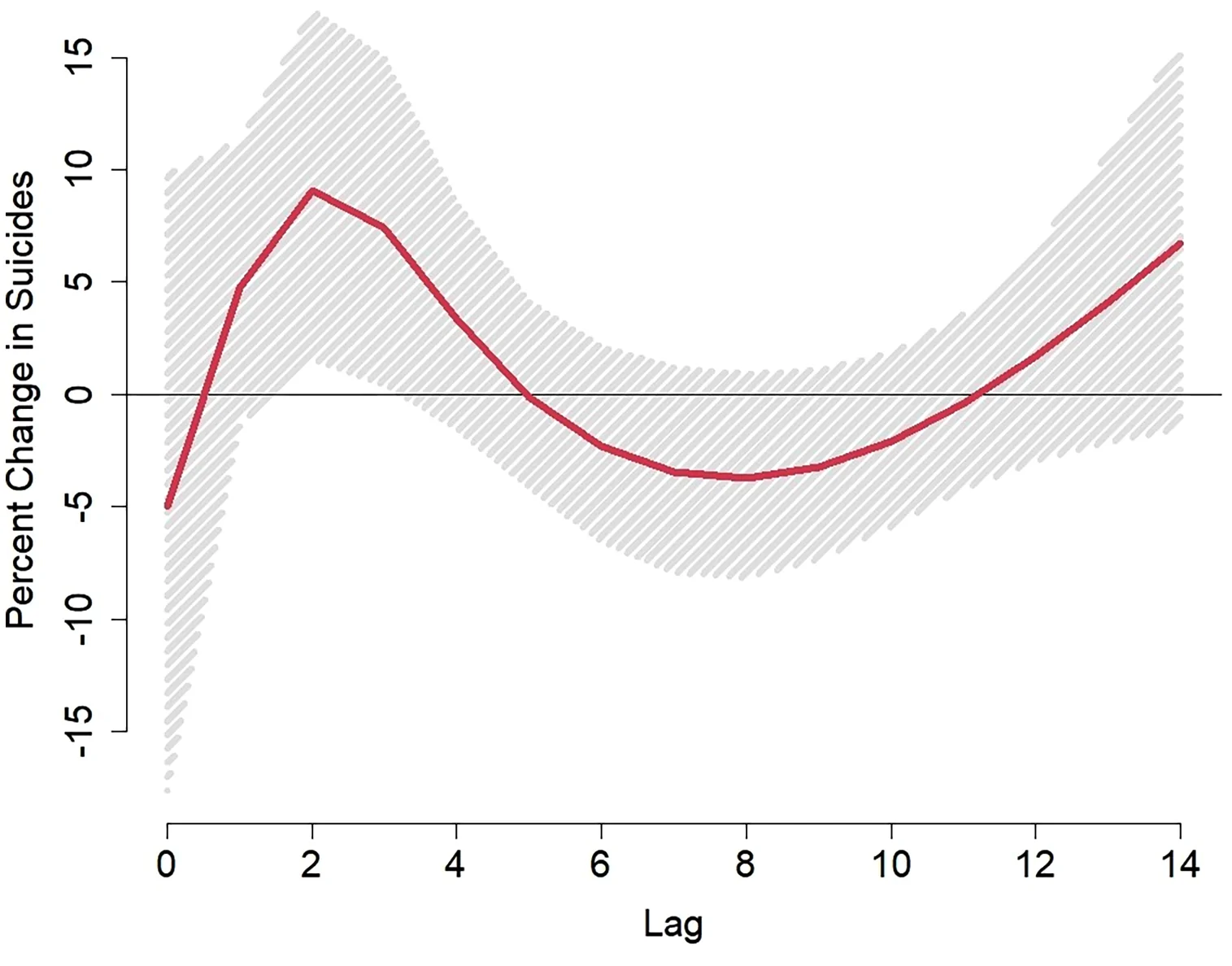
Lag-response curves for the percent change in suicides associated to an extreme heat day in U.S. prisons between 2001 – 2019^a^. ^a^An extreme heat day is defined as a daily maximum temperature above the 90th percentile for the respective prison location.

## References

[1] Skarha J, Spangler K, Dosa D, Rich JD, Savitz DA, Zanobetti A. Heat-related mortality in U.S. state and private prisons: A case-crossover analysis. PLoS One. 2023;18(3):e0281389. doi: 10.1371/journal.pone.0281389 36857338 PMC9976996

