# Supplementary material for: Heat-related mortality in U.S. state and private prisons: A case-crossover analysis
Source: PLoS One. 2023 Mar 1;18(3):e0281389. doi: 10.1371/journal.pone.0281389 (PMC9976996; doi:10.1371/journal.pone.0281389)

**S1 Figure. Association between temperature above prison-specific summer mean temperature and total mortality, heart disease-related mortality, and suicide at the most relevant lag period using a natural cubic spline with two knots**


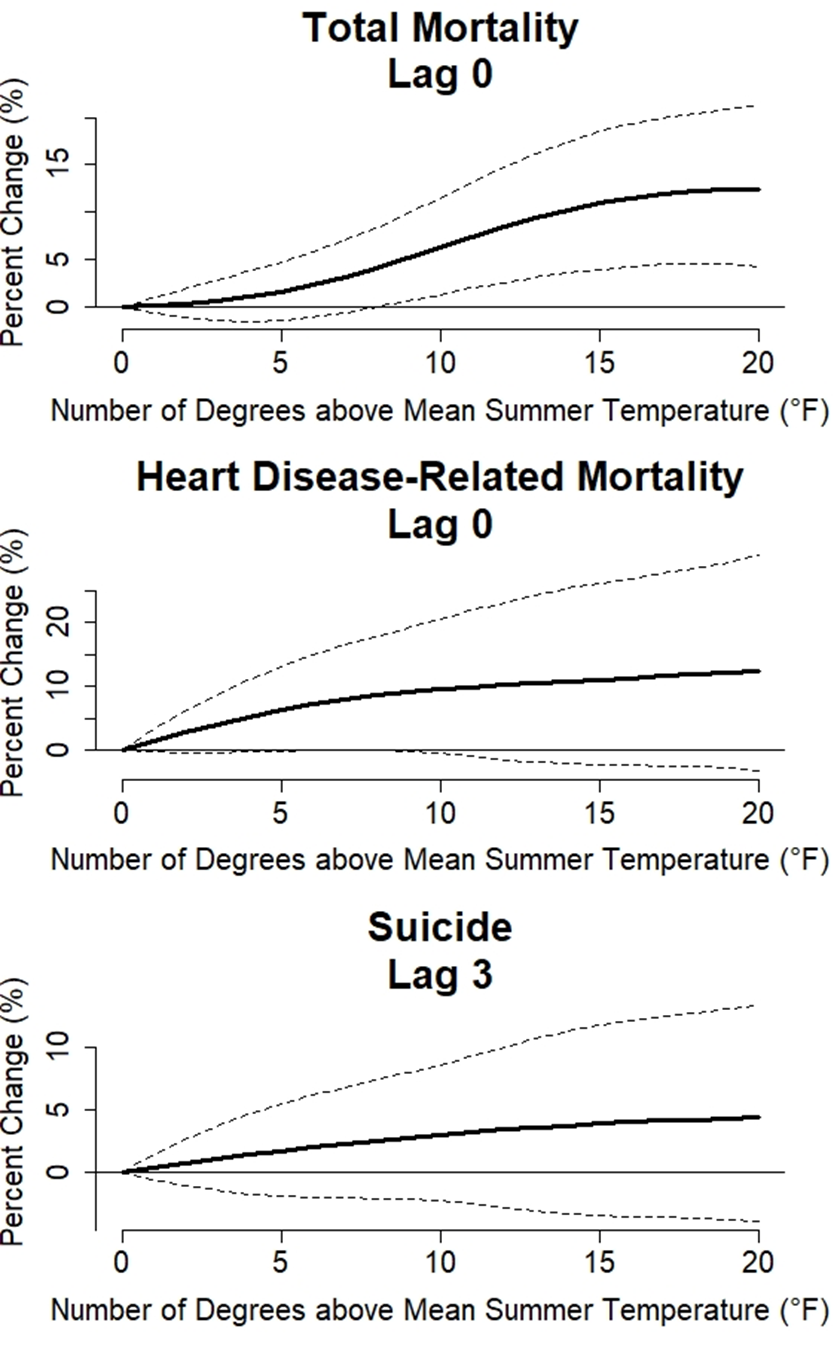

Supplement: S1 Fig — (DOCX) [file pone.0281389.s001.docx]
