# Supplementary material for: Heat-related mortality in U.S. state and private prisons: A case-crossover analysis
Source: PLoS One. 2023 Mar 1;18(3):e0281389. doi: 10.1371/journal.pone.0281389 (PMC9976996; doi:10.1371/journal.pone.0281389)
Supplement: S1 Table — (DOCX) [file pone.0281389.s002.docx]

**S1 Table. Using different temperature metrics with the moving average (Lag01) to model the percent change in total mortality during summer months in U.S. prisons^a,b^**

|  | Continuous Temperature |  |
| --- | --- | --- |
| Change in total mortality using: | 10°F increase in temperature,  % (95% CI) | Non-linear p-value |
| Maximum temperature | 4.8 (0.7, 9.0) | 0.31 |
| Maximum heat index | 2.9 (-0.3, 6.0) | 0.50 |
| Maximum Wet Bulb Globe Temperature | 2.2 (-2.8, 7.0) | 0.62 |

^a^Each temperature variable is centered at the prison-specific summer mean temperature

^b^Non-linear p-value calculate using a natural spline on temperature variable and comparing to model without spline
