# Supplementary material for: Heat-related mortality in U.S. state and private prisons: A case-crossover analysis
Source: PLoS One. 2023 Mar 1;18(3):e0281389. doi: 10.1371/journal.pone.0281389 (PMC9976996; doi:10.1371/journal.pone.0281389)
Supplement: S1 File — (DOCX) [file pone.0281389.s003.docx]

**S1 File. List of medical prison facilities removed from analyses.**

California Medical Facility (CA)

Reception & Medical Center (FL)

Augusta State Medical Prison (GA)

Hospital Galveston (TX)

Carole S. Young Medical Facility (TX)

Corrections Medical Center (OH)

Franklin Medical Center (OH)

Central Medical Unit (NJ)

McCain Correctional Hospital (NC)

Lois M. DeBerry Special Needs Facility (TN)

Iowa Medical & Classification Center (IA)

Bridgewater State Hospital (MA)
