# Supplementary material for: Heat-related mortality in U.S. state and private prisons: A case-crossover analysis
Source: PLoS One. 2023 Mar 1;18(3):e0281389. doi: 10.1371/journal.pone.0281389 (PMC9976996; doi:10.1371/journal.pone.0281389)
Supplement: S2 File — (DOCX) [file pone.0281389.s004.docx]

**S2 File. Regional breakdown of the United States into Northeast (Blue), Midwest (Green), South (Yellow), and West (Red)^a,b,c,d^**


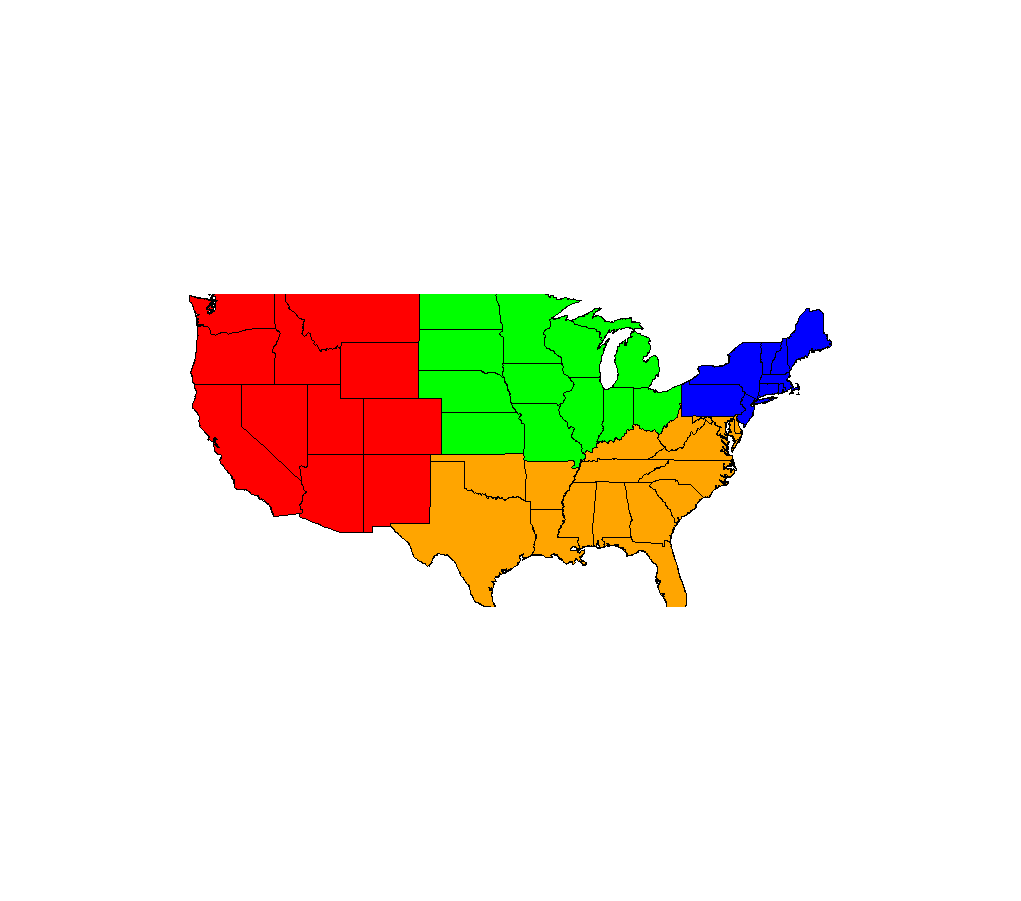


^a^Northeast (Blue) = CT, ME, MA, NH, NJ, NY, PA, RI, VT

^b^Midwest (Green) = IL, IN, IA, KS, MI, MN, MO, NE, ND, OH, SD, WI

^c^South (Yellow) = AL, AR, DE, DC, FL, GA, KY, LA, MD, MS, NC, OK, SC, TN, TX, VA, WV

^d^West (Red) = AZ, CA, CO, ID, MT, NY, NM, OR, UT, WA, WY
