# Supplementary material for: Heat-related mortality in U.S. state and private prisons: A case-crossover analysis
Source: PLoS One. 2023 Mar 1;18(3):e0281389. doi: 10.1371/journal.pone.0281389 (PMC9976996; doi:10.1371/journal.pone.0281389)
Supplement: S1 Appendix — (DOCX) [file pone.0281389.s005.docx]

**S1 Appendix.** **Description of prison facility security level classification.^a^**

Super maximum, maximum/close/high security is characterized by walls or double-fence perimeters, armed towers, or armed patrols. Cell housing is isolated in one of two ways: within a cell block so that a prisoner escaping from a cell is confined within the building or by double security from the perimeter by bars, steel doors, or other hardware. All entry or exit is via trap gate or sally port.

Medium security is characterized by a single or double-fenced perimeter with armed coverage by towers or patrols. Housing units are cells, rooms, or dormitories. Dormitories are living units designed or modified to accommodate 12 or more persons. All entry or exit is via trap gate or sally port.

Minimum or low security is characterized by a fenced or “posted” perimeter. Cell housing units are rooms or dormitories. Normal entry and exit are under visual surveillance.

^a^As defined in the Bureau of Justice Statistics Census of State and Federal Adult Correctional Facilities 2019 questionnaire.

Document: https://bjs.ojp.gov/content/pub/pdf/cj-43a_2019.pdf
