# Supplementary material for: Heat-related mortality in U.S. state and private prisons: A case-crossover analysis
Source: PLoS One. 2023 Mar 1;18(3):e0281389. doi: 10.1371/journal.pone.0281389 (PMC9976996; doi:10.1371/journal.pone.0281389)
Supplement: Attachment — Submitted filename: review.docx [file pone.0281389.s006.docx]

The paper tried to understand the impact of extreme heats on the incarcerated populations using case-crossover analysis. Given the limited number of previous papers focusing on those people, I strongly believe this paper can provide some valuable insights and information. Overall, I really enjoyed reading this manuscript. I raised some major and minor comments. Most of my comments were for clarification. I believe this paper fits well with the PLOS One, and this manuscript can be published after some revisions.

Major comments:

- We considered age at death (< 44, 45 - 54, 55 - 64, > 65), security level of prison facility (low, medium, high), and prison location region in the U.S. (Northeast, Midwest, South, West).
- Please provide any justifications in the introduction why you looked at the impact of security level of prison facility and prison location region in the US. Did you assume that security level is a proxy for stress? I cannot find any reasons why you looked at these variables in the manuscript.
- Can you also provide any map showing your region classification (Northeast, Midwest, South, West)? I know you added some information in the table. But, some audiences outside of the US would have a very limited understanding on this research area. I think showing a map is one of the most intuitive ways.

Minor comments:

- We excluded prisons in mild climate locations where the 90th percentile was less than or equal to 77°F (number of deaths = 262)
- Do you have any specific reasons for selecting 77°F as a threshold.
- We created three categories based on the percentage of the county classified as rural; urban (<10% rural), mostly urban (10% - 50% rural), and rural (>50% rural).
- Did you arbitrarily select these categories?
- Maybe this could be redundant. But, for those who think that NLDAS-2 data already captured urban heat island effects, maybe I would add a sentence showing that urban heat island effects are not captured by NLDAS-2 (Crosson 2020), so we wanted to separately check the impact of urban heat island effects.

Crosson, W. L., Al-Hamdan, M. Z., & Insaf, T. Z. (2020). Downscaling NLDAS-2 daily maximum air temperatures using MODIS land surface temperatures. Plos one, 15(1), e0227480.

- High security prisons and the South region had the highest mortality within their respective categories.
- I would add the actual values (For consistency).
  High security prisons (48.2%) and the South region (47.0%) had the highest mortality within their respective categories.
- Figure 1 caption and figures
- Add what grey area is in the caption
- Add a unit of x-axis (i.e. day)
- Lines 209-212:

This sentence sounds awkward:
Due to our previous findings that temperature-suicide association 209 may also be delayed, Figure 2 shows the suicide lag-response relationship for an extreme heat day using a natural cubic spline.

- (Maybe) Due to our previous findings that temperature-suicide association 209 may also be delayed, we further investigated the suicide lag-response relationship for an extreme heat day using a natural cubic spline.
- And more importantly, I cannot see any reason for doing this analysis (maybe I am wrong). I think Figure 1 and Figure 2 contain the same information. Is there any additional information?
- And where does the number 23% come from? Is this a cumulative percent across lags 1-3?
- From Figure 1 and Table 1, I think most of the impacts are not statistically significant. Only total mortality lag 0 and lag 0-1and total mortality two-day heatwave three-day heatwave are statistically significant. I know you mentioned that some results are not statistically significant (Line 207-208). But, this could mislead audiences that other results excepts them in Lines 207-208 are statistically significant. So, I recommend you first talk about statistically significant results and then introduce other results which are not statistically significant (although not significant, we found some evidence showing ~~~).
- Table 2.

I think most of the relationships are not statistically significant. I think you may need to mention that these results are not statistically significant (e.g. Although mostly not significant, we found some evidence~~ (Line 213~). Some readers may think that all of these results are statistically significant.
